# 

**Published:** 1887-08

**Authors:** 


### Editorial, Etc.

---

### SOUTHERN DENTAL ASSOCIATION.

The Nineteenth Annual Meeting of the Southern Dental Association will be held at the Hygeia Hotel, Old Point Comfort, Virginia, August 30th, 1887.

This meeting will probably be the largest and most enthusiastic gathering of dentists ever held in the United States. Everything possible will be done to make it practical, and therefore profitable.

The Chairman of the Executive Committee and Committee of Arrangements will be in attendance Saturday, August 27th, to see that all the arrangements are perfected.

As there will be a very large attendance, and many arriving at the same time, comfort and convenience will be promoted by notifying Mr. Pike, Manager of the Hygeia, of intended arrival.

The whole of Thursday, and, if necessary, the early morning of each of the other days, before the hour for regular session, will be devoted to Clinics. This feature of the meeting will receive particular attention.

We believe the arrangements will be as near perfect as it is possible to make them.

For the Operative Department we will have nine chairs, with engines, brackets, cases of instruments, gold, and all other necessary appliances, so arranged that the spectators can witness the operations with comfort, without inconvenience to the operators.

All the latest improvements in Electrical Apparatus will be exhibited and demonstrated.

The room for the Prosthetic Department will be fitted up with everything necessary for practical work.

Both rooms are large, light and airy, and in every way admirably adapted for clinical demonstrations.

The Committee of Arrangements will take pleasure in accommodating any member of the profession who may wish to give a clinic, in either Operative or Prosthetic Dentistry, and whose name is not down on the programme.

*Clinical Operators.*—Chas. L. Alexander, Charlotte, N. C., Electric Mallet; W. H. H. Atkinson, New York, Implantation and Sponge Grafting; A. E. Baldwin, Chicago, Immediate Root Filling; W. G. A. Bonwill, Philadelphia, Mallet, Crowns, &c.; A. G. Bouton, Savannah, Ga., Separators and R. D. Clamps; E. Parmley Brown, Flushing, N. Y., Specialties; Wm. Crenshaw, Atlanta, Ga., Electric Mallet and Cohesive Gold; W. B. Finney, Baltimore, Md., Contour and Crown Building; J. H. Harris, Baltimore, Md., Soft Gold and Automatic Mallet; T. May Hunter, Fayetteville, N. C., Gold Filling, Hand Pressure and Mallet; Henry C. Jones, Richmond Va., subject not given; H. J. McKellops, St. Louis, Mo., Herbst's Methods and Appliances; Ed. C. Kirk, Philadelphia, Implantation; C. H. McCowan, Norfolk, Va., Electric Mallet; H. A. Parr, New York, Universal Separators; Safford G. Perry, New York, Separators and Rubber Dam; G. S. Staples, Sherman, Texas, Rubber Dam and Wire Clamps; V. E. Turner, Raleigh, N. C., Soft and Cohesive Gold Filling; S. A. White, Savannah, Ga., Plastic Fillings; G. H. Winkler, Augusta, Ga., Soft Gold

Fillings; E. T. Starr, Philadelphia, Illustrated Diagram of Dental Arch; George Evans, New York, Crown and Bridge Work; W. W. Evans, Washington, D. C., subject not given; D. Genese, Baltimore, Md., Continuous Gum Work; C. P. Grout, New York, Crown and Bridge Work; H. W. Howe, Lawrence, Kansas, Blow Pipe and its work; R. Finley Hunt, Washington, D. C., Celluloid and Gold lined Rubber plates; C. E. Kells, Jr., New Orleans, Modes and Methods with Ideal office, with complete Electric Apparatus; J. Rollo Knapp, New Orleans, Crown and Bridge Work with Cohesive Gold; John H. Meyer, New York, Continuous Gum Work, J. J. R. Patrick, Belleville, Ill., Gold Crowns and Appliances, Rubber Work; Walter R. Starr, Philadelphia, Crown and Bridge Work and Gold Lining; H. A. Parr, New York, Crown and Bridge Work.

STANDING COMMITTEES.

*Education.*—J. H. Coyle, Ga., W. H. Morgan, Tenn; J. H. Durham, N. C.; F. J. S. Gorgas, Md.; J. Y. Crawford, Tenn; G. W. H. Whittaker, Ga.; J. Taft, O.

*Hygiene.*—W. H. Richards, Tenn.; Morgan Adams, Miss.; J. T. Griffith, N. C.; W. J. Barton, Texas; W. D. Dunlap, Ala.; N. A. Teague, Ga.; Wm. B. Pleasants, Va.

*Pathology and Therapeutics.*—W. C. Wardlaw, Ga.; R. B. Winder, Md.; J. H. Prewitt, Ky.; W. J. Reese, Texas; A. T. Bouton, Ga.; G. M. Rosseau, Ala.; R. B. Adair, Ga.

*Histology and Microscopy.*—B. H. Catching, Ga.; D. R. Stubblefield, Tenn.; Geo. H. Winkler, Ga.; T. T. Moore, S. C.; J. L. Fountain, Texas; Chas. L. Steel, Va.

*Chemistry.*—E. S. Chisholm, Ala.; L. P. Dotterer, S. C.; L. G. Noel, Tennessee; Theodore Johnston, S. C.; W. H. Marshall, Miss.; B. H. Douglas, N. C.

*Operative Dentistry.*—M. C. Marshall, Ark., Geo. S. Staples, Texas; Wm. Crenshaw, Ga.; E. E. Kells; La.; E. L. Hunter, N. C.; W. H. Morrison, Mo.; J. W. Scribner, Va.

*Mechanical Dentistry.*—J. Rollo Knapp, La.; W. R. Bull, S. C.; A. H. Hilzim, Miss.; R. R. Freeman, Tenn; J. W. Hunter, N. C.; E. J. De Hart, La.

*Literature and Voluntary Essays.*—B. H. Teague, S. C.;

H. J. McKellops, Mo.; H. M. Grant, Va.; A. E. Baldwin, Ill.; T. H. Parramore, Va.; A. O. Rawls, Ky.; J. R. Patrick, Ill.; W. W. H. Atkinson, N. Y.; E. Parmly Brown, N. Y.; J. B. Hodgkin, D. C.; O. S. Solomon, La.; F. Y. Clark, N. Y.; G. W. Reinhert, Miss.; J. B. Patrick, S. C.; Henry W. Morgan, Tenn.

*Dental Appliances.*—J. S. F. Wright, S. C.; James Johnston, Va., E. M. Allen, Ga., Henry E. Beach, Tenn.; J. F. Thompson, Va.; C. L. Alexander, N. C.; W. S. Carruthers, Texas.

*Publication.*—R. A. Holliday, Ga., J. S. Franklin, Tenn.; W. S. Bacon, Va.; W. B. Finney, Md.; Geo. F. Keesee, Va.; L. M. Cowardin, Va.

The Southern Passenger Association has reduced the fare, to those attending with their families, to one and one third lowest limited rate for the round trip.

All who wish to avail themselves of this liberal concession, should write *at once* to Dr. J. Y. Crawford, 156½ Church St., Nashville, Tenn., for the necessary certificates and instructions, giving the names of those for whom the certificates are required.

This applies to all the roads south of the Ohio and Potomac rivers, and east of the Mississippi.

The roads North and West make the following arrangements:

"Each person attending must purchase a first-class ticket to the place of meeting, for which he will pay the regular fare, and upon request the ticket agent will issue to him a certificate of such purchase.

"If through tickets can not be purchased at the starting point, purchase to the most convenient point where such through tickets can be obtained, and re-purchase through to place of meeting, requesting a certificate from the ticket agent at the point where purchase is made.

"Tickets for the return journey will be sold by the ticket agent at the place of meeting at one-third the highest limited fare, only to those holding certificates signed by the ticket agent at the point where through ticket to the place of meeting was purchased, and countersigned by the Secretary of the Association, certifiying that the holder has been in attendance upon the convention.

"Tickets are good, going, three days before the meeting, and, returning, three days after adjournment."

Parties coming from Texas and Arkansas will apply to Dr. E. S. Jones, Houston, Texas, for certificates.

The social features of such a gathering will not be ignored. The Virginia State Dental Association, at whose invitation we meet in this State, stands pledged to extend a hearty old-fashioned Virginia welcome to all our guests.

The address of welcome will be delivered by Hon. Fitz-hugh Lee, Governor of Virginia.

The meeting will be presided over by W. W. H. Thackston, M. D., D. D. S., the oldest living graduate of Dentistry in the world, who during forty-five years of active practice has been noted for his enthusiastic love of his profession and is still among the most earnest workers for its advancement.

The time has been fixed with special reference to the meeting of the International Medical Congress.

On Saturday, September 3d, a special Steamer will convey the Association and our guests to Washington City, going up the Potomac, one of the loveliest rivers in the world, by day-light, passing Mount Vernon, the home of the "Immortal Washington."

The place of meeting is one of the most delightful resorts on the Atlantic Coast, and if the buildings had been constructed with special reference to such meetings, the arrangements could not have been better.

The manager, Mr. Pike, has generously reduced the fare to those attending the meeting and to members of their families, to $2.50 per day, and promises to "lay Chesapeake Bay and the whole surrounding country under contribution" for our comfort.

Nearly all the Dental Manufacturers in the country have signified tbeir intention to make large display of their goods, and will exhibit everything new in the way of instruments and appliances.

### OFFICERS FOR 1887.

President, W. W. H. Thackston, Virginia; First Vice-

President, B. H. Catching, Georgia; Second Vice-President, J. Rollo Knapp, Louisiana; Third Vice-President, W. H. Richards, Tennessee; Corresponding Secretary, J. Y. Crawford, Tennessee; Recording Secretary, L. P. Dotterer, South Carolina; Treasurer, H. A. Lowrance, Georgia.

*Executive Committee.*—J. Hall Moore, Richmond, Va.; Jos. R. Woodley, Norfolk, Va.; E. S. Chisholm, Tuscaloosa, Ala.

*Committee of Arrangements.*—Jos. R. Woodley, Norfolk, Va., T. H. Parramore, Virginia; G. S. F. Wright. South Carolina.

### *To Readers and Correspondents!*

Communications intended for publication, and exchange journals and papers should be addressed to the EDITOR OF THE AMERICAN JOURNAL OF DENTAL SCIENCE, 845 N. Eutaw St., Baltimore, Md. All letters relating to the business of the JOURNAL, or containing cash remittances, to be directed to Messrs. SNOWDEN & COWMAN. Articles for publication should be received by the editor at least three weeks previous to the first month on which the number in which it is designed for them to appear, is issued.

☞ The AMERICAN JOURNAL OF DENTAL SCIENCE will contain fifty pages of reading matter in each number, making a volume of about *Six Hundred* pages,

EXCLUSIVE OF ADVERTISEMENTS.

